# Correction: Covalent organic nanosheets for bioimaging

**DOI:** 10.1039/c8sc90216j

**Published:** 2018-10-31

**Authors:** Gobinda Das, Farah Benyettou, Sudhir Kumar Sharma, Thirumurugan Prakasam, Felipe Gándara, Victor A. de la Peña-O’Shea, Na’il Saleh, Renu Pasricha, Ramesh Jagannathan, Mark A. Olson, Ali Trabolsi

**Affiliations:** a Chemistry Program , New York University Abu Dhabi (NYUAD) , United Arab Emirates . Email: at105@nyu.edu; b Engineering Division , New York University Abu Dhabi (NYUAD) , United Arab Emirates; c The Materials Science Factory , Instituto de Ciencia de Materiales de Madrid - CSIC , Sor Juana Inés de la Cruz 3 , 28049 Madrid , Spain; d Photoactivated Processes Unit , IMDEA Energy , Avd. Juan de la Sagra 3 , Mostoles 28935, Madrid , Spain; e United Arab Emirates University , College of Science , Department of Chemistry , Al-Ain , United Arab Emirates; f School of Pharmaceutical Science and Technology , Health Science Platform , Tianjin University , 92 Weijin Rd. Nankai, District , Tianjin 300072 , P. R. China

## Abstract

Correction for ‘Covalent organic nanosheets for bioimaging’ by Gobinda Das *et al.*, *Chem. Sci.*, 2018, DOI: ; 10.1039/c8sc02842g.



## 

In the original article, the name of one of the authors, Sudhir Kumar Sharma, was displayed incorrectly. The correct presentation of this name is as shown above.

The Royal Society of Chemistry apologises for these errors and any consequent inconvenience to authors and readers.

